# A modified Lichtenstein hernia repair using fibrin glue

**DOI:** 10.4103/0972-9941.27724

**Published:** 2006-09

**Authors:** Giampiero Campanelli, Diego Pettinari, Marta Cavalli, Ettore Contessini Avesani

**Affiliations:** Department of Surgical Sciences, University of Milano, Policlinico Hospital I.R.C.C.S, Pad. Beretta Est, Milano, Italy

**Keywords:** Chronic pain, fibrin glue, lichtenstein hernia repair

## Abstract

In recent years, general surgeons who perform inguinal hernia repair have paid attention to successful reduction in the recurrence rate. The Lichtenstein technique is widely used because it is easy to learn and is associated with a low rate of complication and recurrences. Today, the new objective in primary hernia surgery should be to reduce complications such as chronic pain. Chronic pain after hernia repair can be disabling, with considerable impact on quality of life and there is evidence to suggest increased use of health services by patients who have chronic pain. We have proposed an international randomized controlled trial with seven referenced European centers: The TI.ME.LI. trial. The aim of this study is to evaluate pain and further disabling complications in patients undergoing Lichtenstein technique for primary inguinal hernia repair by fixing the mesh with fibrin sealant versus sutures (control group).

In recent years, general surgeons performing inguinal hernia repair have paid attention to successful reduction of recurrence rates. The Lichtenstein technique is widely used because it is easy to learn and it is associated with a low rate of complications and recurrences.[1] Meshoma and complications related to the migration of the plug and mesh are a problem, but their incidence is low. These are more frequently encountered after laparoscopic hernia repair.[2] Today, the new objective in primary hernia surgery should be to reduce disabling complications such as chronic pain.

Inguinal hernia repair is a common surgical procedure performed worldwide, with an annual procedural rate of 2,800 per million people in U.S. alone.[3] In U.K. about 80,000 primary inguinal herniorrhaphys were performed in National Health Service hospitals[4] and in Italy the number is about 150,000 per year.

Inguinal herniorrhaphy is often performed as a day-case procedure with minimal postoperative morbidity. After inguinal hernia repair, patients can return to work early and enjoy a good quality of life.[5] Despite the fact that recent meta-analyses have suggested that laparoscopic surgery is associated with less postoperative pain and more rapid return to normal activity,[6,7] open-mesh repair is recommended by National Institute for Clinical Excellence (N.I.C.E.)[4] and by some authors,[8] as well as national guidelines. Most studies include postoperative pain as an outcome measure. A little pain in the postoperative period is always expected and requires appropriate analgesia. Chronic pain after hernia repair can be disabling, with considerable impact on quality of life[9,10] and there is evidence to suggest increased use of health services by patients who have chronic pain.

Chronic pain or persistent neuralgia has been recognized as a complication after inguinal hernia repair but was reported in the 1980s as a rare and infrequent condition.[11] Studies from the mid-1990s have reported a higher frequency of patients reporting pain after hernia repair more than 1 year after surgery.[12–14] Estimates of chronic pain vary considerably from 0 to 53%.[5,15] It can be mild to severe, even disabling and can adversely affect quality of life.[16]

The ‘pain complex syndrome’ after hernia repair includes three different aspects: 1) numbness and burning sensation (hypoesthesia, hyperesthesia and paresthesia) - the incidence was observed in 10 to 23% in open inguinal hernia repair in Cochrane review;[17] 2) groin discomfort - its incidence is reported in most studies to be from 11 to 27%;[18,19] 3) neuralgia,[15,20] with radiation of pain to the skin of the corresponding hemiscrotum, labium majus and Scarpa's triangle. Consensus of European Hernia Society, in TI.ME.LI. trial, has defined pain complex syndrome as the presence of one of the three or all of these aspects 1 year after surgery. In terms of percentage, the incidence of pain complex syndrome is assumed to be about 25% of patients undergoing inguinal hernia repair.

Etiology of this problem includes non-neuropathic and neuropathic causes or a combination of both. Non-neuropathic causes include mechanical pressure of folded or wadded mesh, periosteal reaction and scar-tissue formation. Neuropathic pain can be caused by compression of one or more nerves by ‘perineural fibrosis,’ suture material, staples and tacks or by nerves injuries. So if it is possible to limit the use of suture and fixation devices, chronic groin pain could be reduced.[21,22] Following this idea, we have started to perform an observational study about sutureless glue mesh repair for uncomplicated primary inguinal hernia, for not selected patients, in one of the hospitals where we normally work.

Based on this study, we have planned an international randomized controlled trial with seven referenced European centers: the TI.ME.LI. trial.

## MATERIALS AND METHODS

Our observational study design focuses on the absence of complications like pain complex syndrome in the patients undergoing hernia repair with a totally sutureless technique and a mesh fixation with fibrin sealant.

This study took place from June 2004 to March 2006. A total of 113 consecutive patients (101 males [89.4%] and 12 females [10.6%]) were operated with this technique for primary unilateral inguinal hernia. The age range varied from 45 to 65 years for females (mean age 55) and from 26 to 90 years for males (mean age 58). Exclusion criteria were recurrent hernia, femoral hernia, complicated hernia, obesity. All surgeries were performed by the same most experienced surgeon under local anesthesia. All patients were fully briefed about the surgical procedure and an informed consent was obtained.

The median operating time (skin-to-skin) was 40 min and no immediate postoperative complications were observed. All patients were operated as day cases, with a maximum hospital stay of 20 h; none required readmission.

### Technique

The inguinal region was prepared and the hernia sac managed according to the Lichtenstein technique.[23] The ilioinguinal nerve, the iliohypogastric nerve and the genital branch of the genitofemoral nerve were identified and preserved. An 8 × 15 cm polypropylene mesh tailored to the individual patient was placed on the inguinal floor with a pubic overlay of about 2 cm and fixed to the aponeurotic tissue above pubic tubercle with a glue spot. Then, the mesh was placed on the inguinal canal and glued to the inguinal ligament and to the internal oblique muscle. The glue used was fibrin glue, around 1 ml being required for fixation. The two components of the glue were mixed during the operation to form fibrin and applied with a spray device, ensuring a complete covering of mesh on the floor of the inguinal canal. After placing the split mesh around the spermatic cord, a single stitch of a nonre-adsorbable material such as polypropylene was used to fix the two tails and the aponeurosis was closed with an absorbable running suture. The subcutaneous and cutaneous layers were approximated with an absorbable running suture.

### The TI.ME.LI. trial

Based on our hypothesis and on this observational study, we have proposed a randomized controlled trial, according with the nations involved and our own investigators:

- Prof. Giampiero CAMPANELLI, Milan, Italy (principal investigator)
- Prof. Gérard CHAMPAULT, Bondy, France
- Prof. Manuel HIDALGO, Madrid, Spain
- Dr. Andreas HOEFERLIN, Mainz, Germany
- Prof. Marc MISEREZ, Leuven, Belgium
- Prof. Andrew KINGSNORTH, Plymouth, UK
- Prof. Jacob ROSENBERG, Copenhagen, Denmark

The TI.ME.LI. trial is a prospective randomized controlled trial - a patient- and evaluator-blinded study. The aim of this study is to evaluate pain and further disabling complications in patients undergoing Lichtenstein technique for primary inguinal hernia repair by fixing the mesh with fibrin sealant versus sutures (control group).

Based on review of literature[5,15,16] and personal experience, we assume a prevalence of 25% of ‘complex pain syndrome’ in the control group. So, applying the total tension-free technique with glue fixation, we expect to reduce this to at least 12.5% of chronic pain, numbness or groin discomfort. The sample size was calculated and 328 adult males divided into two equal groups requiring a unilateral tension-free hernioplasty by Lichtenstein technique will be included in the study. Patients will be stratified by the study center.

In the fibrin sealant group, the two tails of the mesh will be fixed together by overlapping their edges and by surrounding the cord. The tails will be joined up to by one suture. No other sutures will be used. First, the mesh will be put correctly in place. A small spot (1 ml) of glue will be applied on the pubis under the mesh without spray and then the remaining part (3 ml) over the mesh on the entire surface in a thin uniform layer by spray. Four milliliters of fibrin sealant will be used per mesh. In the control group, the mesh will be fixed in a conventional manner. Nerve resection (if occurred) will be recorded; it should be avoided.

A close follow-up will be performed (1 week, 1 month and 6 months) after surgery, with a final evaluation 12 months after surgery, which will end the participation to the study. Visual analog pain scores and quality of life (S.F.12) scores will be used to evaluate the results from the patients. The following parameters will be evaluated as secondary endpoints: recurrence; overall wound-healing complication rate (bleeding complications, bruising seroma, wound infection, mesh infection); early postoperative pain at 1 week and 1 month after surgery; mid-term postoperative pain at 6 months after surgery; incidence of patients without pain at 1, 6, 12 months after surgery; use of analgesic drugs; patient's satisfaction; incidence of adverse events; quality of life assessed by a questionnaire preoperatively, at 1, 6 and 12 months; hospital stay; and time to return to normal activities.

Inclusion criteria for the study are:

- Active males over the age of 18 years and up to 70 years.
- Presence of an uncomplicated unilateral primary inguinal hernia.
- Subjects eligible for elective inguinal hernia repair using Lichtenstein technique.
- Written signed informed consent.

Subjects fulfilling the following exclusion criteria will not be recruited into the study:

- Recurrent, bilateral, scrotal, incarcerated or femoral hernias.
- Hernia types L3 and M3 according the European Hernia Society classification assessed preoperatively or intra-operatively.
- BMI equal to or more than 35.
- Concomitant abdominal surgery.
- Ongoing long-term analgesic or steroid treatment
- Patients receiving antiplatelet agents or anticoagulants.
- Known history of alcohol or drug abuse
- Liver cirrhosis (child C).
- Hypersensitivity to bovine aprotinin or known immunodeficiency.
- Severely compromised physical or psychological health, concurrently participating in another clinical trial and having received another investigational drug or device within the last 30 days.

At the moment, the trial is underway in all the seven countries and the final report is expected in the 2008 spring.

The analysis of the primary endpoint will be performed on two analysis sets - ‘Intent to Treat’ population and ‘per protocol’ population. The percentage of patients with at least one of the complications defined and its 95% confidence interval will be described per group and in total. It will be compared between the two groups using the Chi-square test.

An Independent Data Monitoring Committee has been established:

- Prof. Volker Schumpelick, Germany.
- Prof. Jean-Bernard Flament, France.
- Prof. Franco Corcione, Italy.

## RESULTS

We have a good evaluation of our observational study in the patients operated, 18 months after surgery. No complications like hematoma or seroma were observed in follow-up at 1 week, 1 month, 6 months and 18 months. At 18 months, none of the patients had developed a recurrence. In our observational study, postoperative pain was evaluated by a phone call from 1 week to 18 months after surgery, using a score going from 1 to 5, where 1 corresponded to a pain-free status and 5 to the worst conceivable pain.

Of the total 113 patients, 92 patients (81.5%) - 82 male and 10 female - reported complete absence of pain. Eighteen patients (15.8%) - 16 male and 2 female - reported a pain score of 2 and only 3 male (2.7%) patients reported a pain score of 3. In no patients was a score of more than 3 registered [Table 1].

**Table 1:** Postoperative pain scores

Pain evaluation	Male	Female	Total
1	82	10	92
2	16	2	18
3	3	-	3
4	-	-	-
5	-	-	-

The objective of the study was to evaluate the mid- and long-term postoperative pain, numbness and groin discomfort in open inguinal hernia repair by Lichtenstein technique after mesh fixation with fibrin sealant compared with mesh fixation with sutures.

## DISCUSSION

For a common pathology such as inguinal hernia, the goal is to have ‘no defects.’ Thus for primary inguinal hernia by Lichtenstein's operation, we should aim for the following: a) local anesthesia; b) mesh (and plug?); c) tension-free sutureless; d) patients are able to anbulate early and get discharged the same day; e) rapid return to work; f) a rcurrence rate of < 1%; and g) absence of patient discomfort, numbness or neuralgia.

Our preliminary observational study has shown that the use of fibrin sealant in inguinal hernia repair protects the patients from groin discomfort for as long as 18 months. So mesh fixation with fibrin sealant appears to be suitable for use in open tension-free repair. At 18 months, we have not observed any complications related to the technique.
